# A new vetulicolian from Australia and its bearing on the chordate affinities of an enigmatic Cambrian group

**DOI:** 10.1186/s12862-014-0214-z

**Published:** 2014-10-21

**Authors:** Diego C García-Bellido, Michael S Y Lee, Gregory D Edgecombe, James B Jago, James G Gehling, John R Paterson

**Affiliations:** School of Earth and Environmental Sciences & Environment Institute, University of Adelaide, Adelaide, SA 5005 Australia; Earth Sciences Section, South Australian Museum, North Terrace, Adelaide, SA 5000 Australia; Department of Earth Sciences, The Natural History Museum, Cromwell Road, London, SW7 5BD UK; Barbara Hardy Institute, School of Natural and Built Environments, University of South Australia, Mawson Lakes, SA, 5095 Australia; Division of Earth Sciences, School of Environmental and Rural Science, University of New England, Armidale, NSW 2351 Australia

**Keywords:** Deuterostomes, Chordata, Vetulicolia, Tunicata, Cambrian, Emu Bay Shale

## Abstract

**Background:**

Vetulicolians are one of the most problematic and controversial Cambrian fossil groups, having been considered as arthropods, chordates, kinorhynchs, or their own phylum. Mounting evidence suggests that vetulicolians are deuterostomes, but affinities to crown-group phyla are unresolved.

**Results:**

A new vetulicolian from the Emu Bay Shale Konservat-Lagerstätte, South Australia, *Nesonektris aldridgei* gen. et sp. nov., preserves an axial, rod-like structure in the posterior body region that resembles a notochord in its morphology and taphonomy, with notable similarity to early decay stages of the notochord of extant cephalochordates and vertebrates. Some of its features are also consistent with other structures, such as a gut or a coelomic cavity.

**Conclusions:**

Phylogenetic analyses resolve a monophyletic Vetulicolia as sister-group to tunicates (Urochordata) within crown Chordata, and this holds even if they are scored as unknown for all notochord characters. The hypothesis that the free-swimming vetulicolians are the nearest relatives of tunicates suggests that a perpetual free-living life cycle was primitive for tunicates. Characters of the common ancestor of Vetulicolia + Tunicata include distinct anterior and posterior body regions – the former being non-fusiform and used for filter feeding and the latter originally segmented – plus a terminal mouth, absence of pharyngeal bars, the notochord restricted to the posterior body region, and the gut extending to the end of the tail.

**Electronic supplementary material:**

The online version of this article (doi:10.1186/s12862-014-0214-z) contains supplementary material, which is available to authorized users.

## Background

The enigmatic Cambrian vetulicolians have been allied to various protostome and deuterostome animal lineages over the decades since their discovery. They were initially described as bivalved arthropods because their bipartite body was interpreted as an anterior carapace with a segmented abdomen extending beyond the carapace margins [1,2]. No segmental appendages have been found in any vetulicolian, and closest affinities to deuterostomes were instead proposed because of the presence of gill slits, a purported mesodermal skeleton and a possible endostyle [3,4]. In this context, vetulicolians were suggested to be either stem-group chordates [4] or crown-group chordates most closely allied to tunicates [5]. Phylogenetic analyses coding for characters in all known vetulicolians using both protostome and deuterostome taxa [6] did not resolve the systematic position of the group, but suggested kinorhynchs and tunicates to be likely relatives under either a protostome or a deuterostome model, respectively.

The presence of pharyngeal gill slits, pouches, plus possible circular and longitudinal body-wall musculature ally vetulicolians with deuterostomes [7-10], but their systematic position relative to the crown-group phyla has remained uncertain. The monophyly of Vetulicolia has been regarded as an open question [8], and most debate discusses whether they are either stem-group deuterostomes [8,9] or stem-group chordates [11]. Vetulicolia is now known from 14 species classified as nine genera, four families and two orders (Additional file 1). Most species have been described from Cambrian Series 2, Stages 3 and 4 in South China (the Chengjiang and Guanshan biotas), supplemented by taxa from Sirius Passet (Stage 3) in Greenland and the Burgess Shale (Stage 5) in western Canada.

Abundant, well-preserved material of a new vetulicolian from the early Cambrian of Australia extends the group’s distribution to East Gondwana, and a new phylogenetic analysis provides evidence that Vetulicolia are crown-group chordates, most closely related to tunicates.

## Results

### Systematics

This published work and the nomenclatural acts it contains have been registered in Zoobank: http://zoobank.org/urn:lsid:zoobank.org:pub:D9915A7D-DD12-4031-AA36-D1879A96B6D0.

Class Vetulicolida Chen & Zhou, 1997.

*Nesonektris aldridgei* gen. et sp. nov.

#### LSID

urn:lsid:zoobank.org:act:31858511-031A-4098-9852-18B42757C847.

#### Etymology

*Nesos* (f.), Greek for island; *nektris* (f.), for swimmer. Specific epithet in memory of Dick Aldridge, who led a pioneering effort to resolve vetulicolian affinities.

#### Holotype

**Figure 1:**
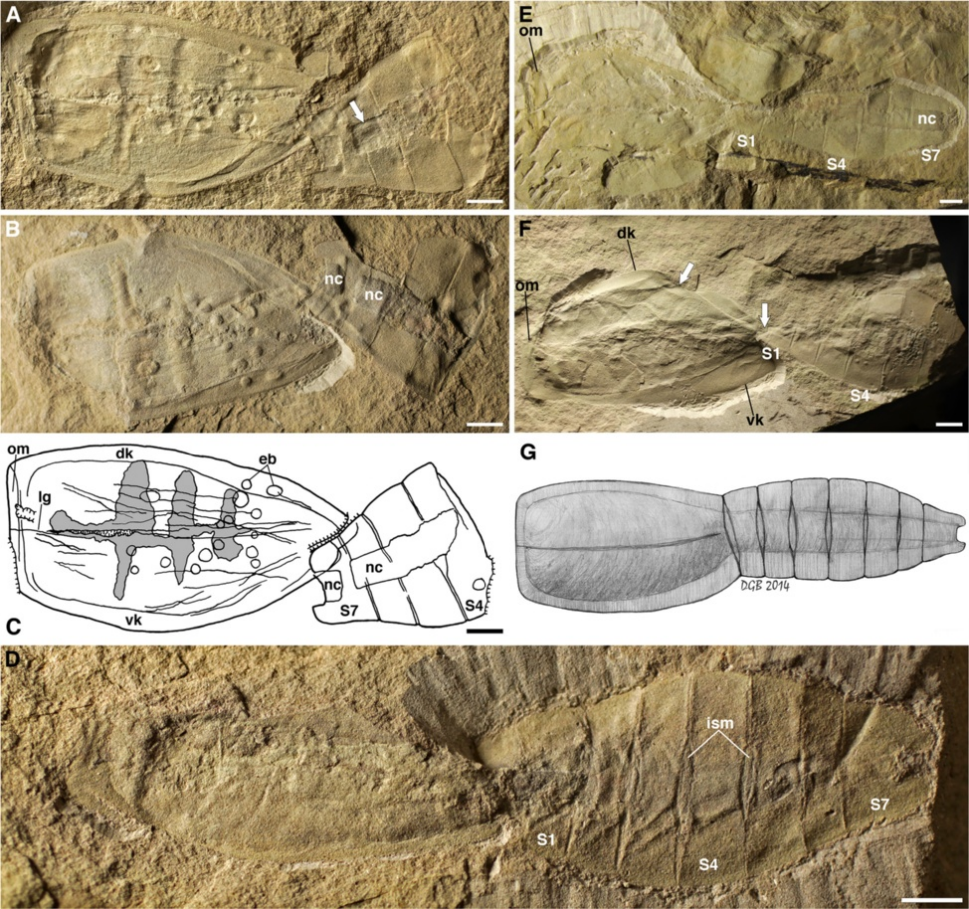
**The early Cambrian vetulicolian** ***Nesonektris aldridgei*** **. A**–**C**, Holotype SAM P45212a,b, specimen with distal end of posterior body region (S4–S7) folded over itself, anterior towards left; **A**, part, anterior rim exposed by preparation (arrow points to cuticle wall under the rod); **B**, Counterpart, with severed notochord (detail in Figure 2B); **C**, camera lucida drawing, grey indicates sediment infill; **D**, SAM P49084a, nearly complete specimen, only lacking one side of anterior region body; **E**, SAM P48073a, nearly complete specimen; **F**, SAM P48015a, nearly complete but folded specimen; showing fold on anterior region (oblique arrow) and continuous connection between anterior and posterior body regions along the dorsal margin (vertical arrow); **G**, Reconstruction; abbreviations: dk = dorsal keel; eb = epibionts; ism = intersegmental membrane; lg = lateral groove; nc = notochord; om = oral margin; vk = ventral keel; S1–S7 = posterior body region segment number; scale bars, 5 mm.

**Figure 2:**
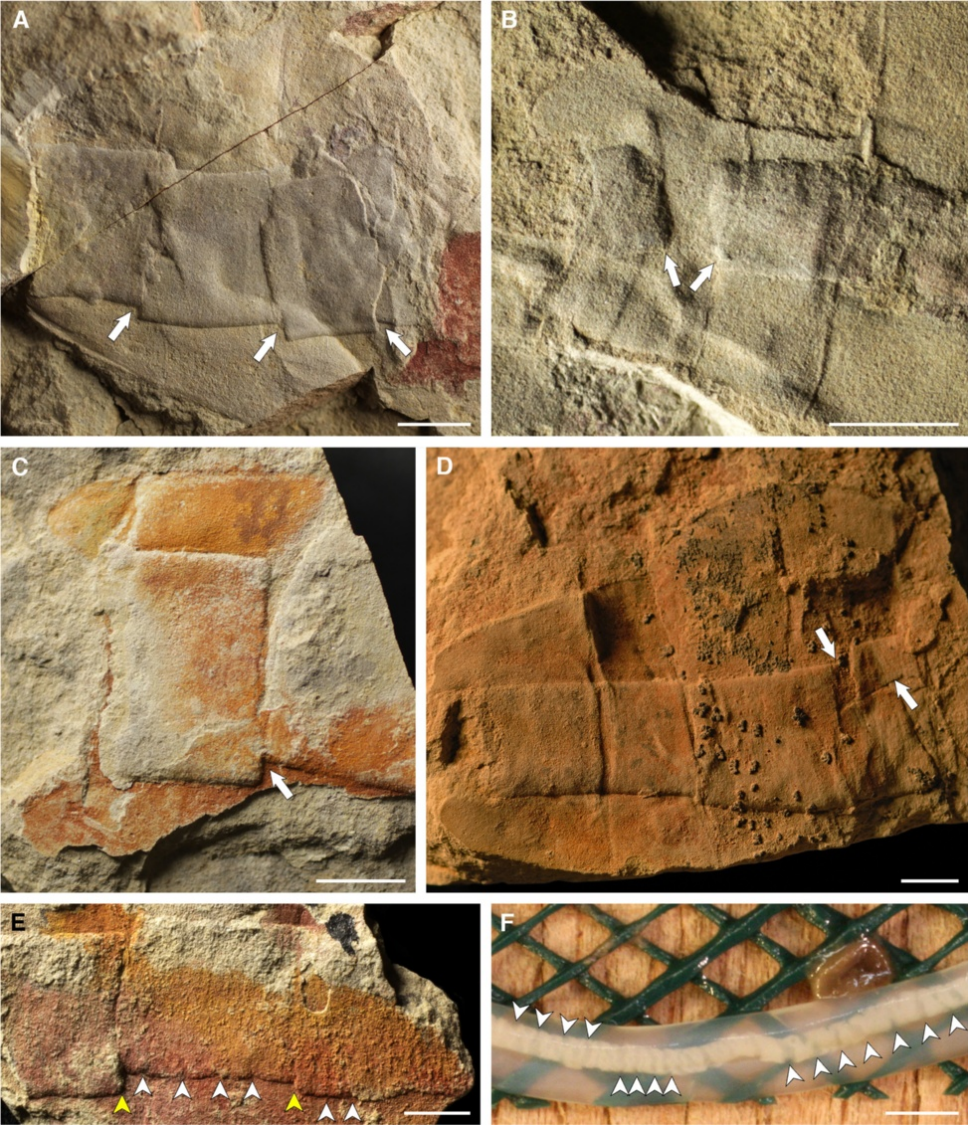
**Details of axial rod (interpreted as notochord) of** ***Nesonektris aldridgei*** **. A**, SAM P46336a, detail of four offset notochord fragments overlapping each other; **B**, Holotype SAM P45212 (see Figure 1B), detail of displaced notochord at posterior end of body; **C**, SAM P47166, detail of notochord with detached terminal block; **D**, SAM P45215, posterior end of specimen with notochord displaced in S4; **E**, SAM P43655a, detail of displaced notochord, with small offsets at regular intervals (white arrowheads) corresponding to discs and larger displacement into blocks of discs (yellow arrowheads); **F**, partially decayed hagfish notochord, showing displacement of discs, image courtesy of Robert Sansom; scale bars, 5 mm **(A–E)** and 2 mm **(F)**.

SAM P45212a,b (Figure 1A-C, Figure 2B).

#### Referred material

Fifteen figured paratypes and c. 150 additional unfigured specimens (Additional file 2).

#### Locality and horizon

Buck Quarry, the wave-cut platform and coastal cliffs, Big Gully, Kangaroo Island, South Australia; Emu Bay Shale, *Pararaia janeae* Zone (Cambrian Series 2, Stage 4).

#### Diagnosis

Large vetulicolian with anterior and posterior body regions subequal in length. Anterior region subquadrate, with vertical oral margin connected to dorsal and ventral keels. Narrow lateral groove present, but no evidence of gill pouches. Anterior and posterior regions connected mid-dorsally at an angle. Elongate posterior region of seven segments, tapering after the fourth; terminal segment longer than others, ending in two lobes connected by a flat notch. Internal rod-like structure extends along axis of posterior body region.

#### Description

**Figure 3:**
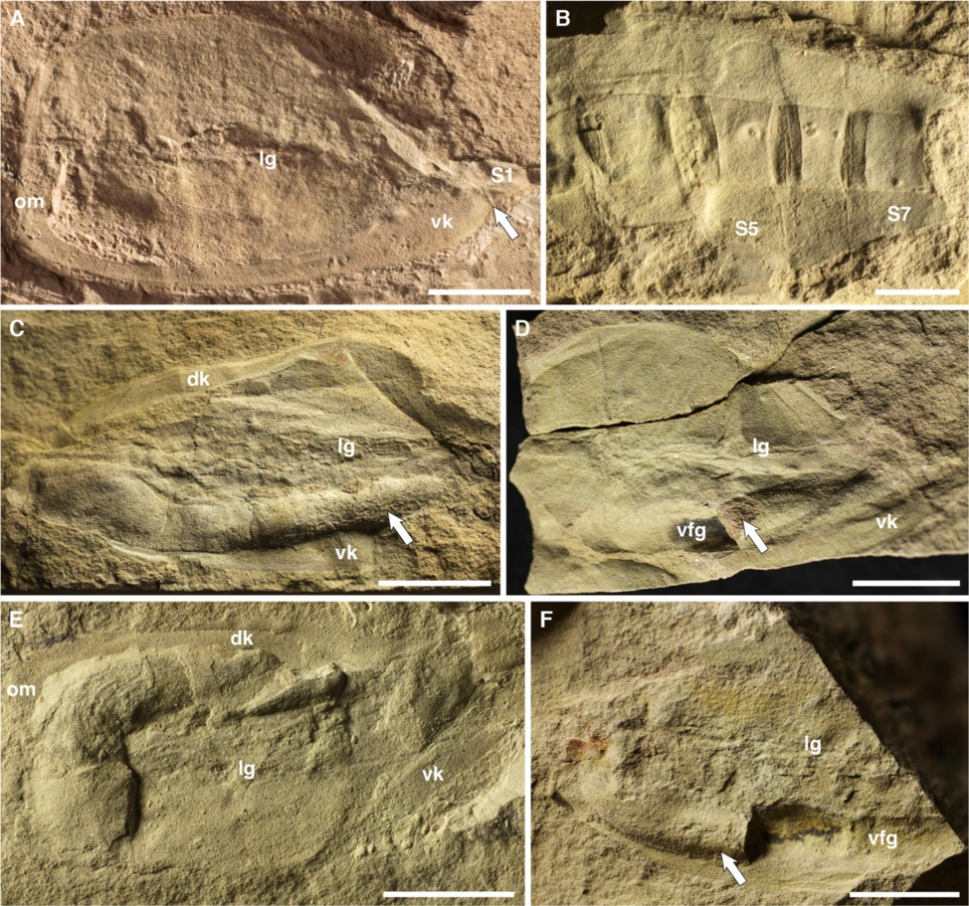
**Anterior and posterior body regions of the early Cambrian vetulicolian** ***Nesonektris aldridgei*** **. A**, SAM P49080a, showing the joint between the anterior region and the first segment of the posterior region (arrow), in its ventral area; notice the angle of the joint (see Figure 1G); **B**, SAM P49662a, specimen with intersegmental membranes imprinted on partially mineralized axial rod due to compaction during burial; **C**, SAM P47152a, showing remains of food boluses (arrow) in ventral food gutter (sensu Ou et al. [9], Figure six A-E); **D**, SAM P47168b, with ventral food gutter; **E**, SAM P49076a, anterior region partially infilled with sediment; **F**, SAM P48105a, with ventral food gutter; additional abbreviations: vfg = ventral food gutter; figs **D** and **F** are mirror images of the original to aid with comparison with main figures in text (posterior to right); scale bars, 10 mm.

**Figure 4:**
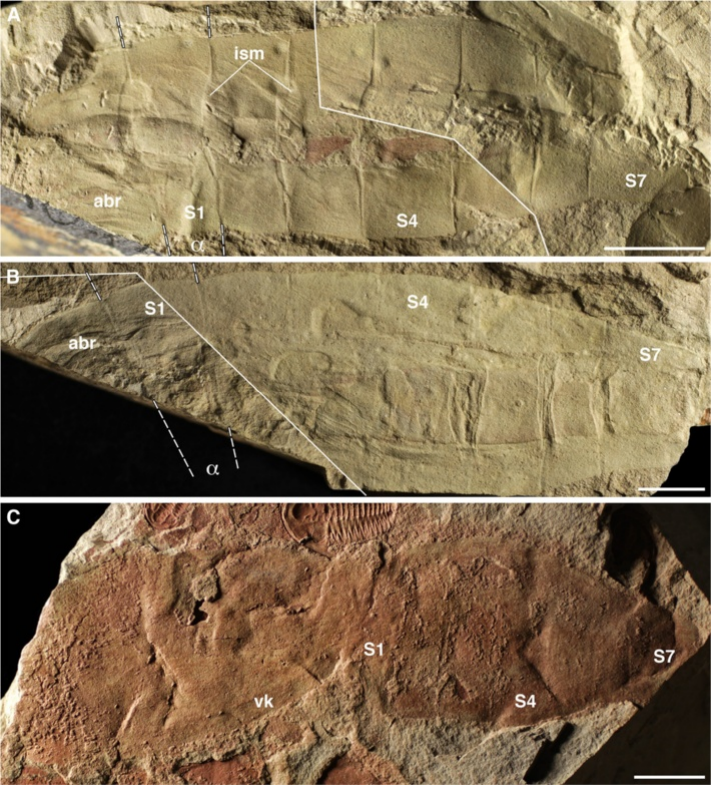
**Posterior body regions of the early Cambrian vetulicolian** ***Nesonektris aldridgei*** **. A**, SAM P48093a,b part and counterpart, composite image; **B**, SAM P48013a,b part and counterpart composite image; **C**, SAM P49147a; mirror images have been used for **A** and **B** to aid with comparison with main figures in text (posterior to right); additional abbreviations: α = angle of S1 segmental boundaries; abr, anterior body region; solid lines indicate limits of part and counterpart in composite images, while dashed lines indicate S1 segmental boundaries; scale bars, 10 mm.

Anterior and posterior body regions are often found disarticulated, with the most complete specimen 125 mm in length. Extrapolation from incomplete specimens, however, suggests maximum sizes of over 170 mm in length. The anterior region is up to 70 mm long and 42 mm high, subquadrate anteriorly and slightly dorso-ventrally asymmetrical, tapering backwards. An oral marginal zone is of constant width, extending dorsally and ventrally into marginal keels that widen posteriorly, especially the ventral one, which reaches the axial posterior region. The anterior region is generally smooth, with some specimens showing a dimpled surface, and occasional wrinkles due to burial and compaction of the cuticularized body-wall (Figure 1C). A thin lateral groove (Figures 1A, C, G, 3A, C-F) extends longitudinally from the oral marginal zone to the posterior portion of the anterior body, but no openings (slits, gills or pouches) can be recognized in association. The internal cavity is occasionally filled by sediment (Figures 1A-C, 3E), and several specimens (Figure 3C, D, F) preserve a ventral food gutter (compare [9], their Figures six A, D, E). The posterior region (tail) is up to 90 mm long and 40 mm high, and displaced towards the dorsal part of the animal (Figure 1G), but not as much as in *Vetulicola* ([9], their Figure seven A). It is divided into 7 segments, connected by intersegmental membranes that become shorter and narrower distally (Figures 1D, G, 4). The anterior and posterior boundaries of the first segment (S1) are at an approximate angle of 10–15° to one another, so S1 is narrower ventrally (Figures 4A, B, but also 1D, G). Length and width slightly increase caudally up to S4 and taper after S5. S6 is consistently the shortest, while S7 is the longest but narrowest dorso-ventrally, tapering considerably and presenting a distal notch. Dorsal flanges are present in all tail segments.

A three-dimensional axial rod-like structure is restricted to the posterior region of *Nesonektris.* It has a relatively constant width of about 20% the width of the whole tail – including the dorsal and ventral flanges – but fills a high proportion of the tail cavity. It reaches the distal end of the posteriormost segment, terminating in the caudal notch. It occasionally shows partition into sections that are offset or separated and frequently decoupled from the posterior segmentation of the body (Figure 2).

## Discussion

### Interpretation of the rod-like structure: Notochord, gut or coelomic cavity?

We are doubtful about an identity of this axial rod as a gut, a structure that has been identified in several other vetulicolians [12]. A gut was shown, for example, by Caron [13] in some specimens of *Banffia* (Figure twenty F-I), by Hou [1] in *Didazoon* (Figures one A, D and five A), and by Aldridge et al. [6] in *Vetulicola* (Plate one, Figures six to eight) and *Pomatrum* (Plate 4, Figures eight and twelve). The gut in these taxa is represented by a typically narrow tube with continuous margins that originates in the anterior body region (and is sometimes associated with possible food boluses [5]); this tube can bulge and constrict along its length (see Aldridge et al. [6], e.g., Figure six H, I) and is often coiled (e.g., Hou [1], Figure five A, B; Hou and Bergström [2], Plate one, Figure eight; Chen and Zhou [14], Figure fourteen A), with its contents occasionally extending into the matrix beyond the terminal anus (Ou et al. [9], Figure six H). In contrast, the larger, rod-like structure in *Nesonektris* has a more constant width, is only present in the posterior body region and never extends beyond the distal end of that region. Most notably, its partitioning into sections that are block-like and offset (Figure 2) is inconsistent with a gut in general or the known gut of vetulicolians in particular. It is situated medio-dorsally in the tail rather than ventrally (the latter being expected for a gut). This structure is never found to be continuous with food boluses in the anterior body region (Figure 3C, D, F); in a specimen where they appear aligned they are in fact on different levels (Figure 1D). A specimen of *Vetulicola* from Chengjiang also shows a rod that closely resembles this axial structure and was interpreted as a possible notochord (Aldridge et al. [6], Plate one, Figures five and nine). The posterior body region of other specimens of *Vetulicola* (Chen and Zhou [14], Figure fourteen A, B) shows a coiled gut superimposed on an axial structure similar to that of *Nesonektris*, indicating that they are two distinct structures and not taphonomic variants of the same feature. This superposition can be easily explained in the better preserved specimen illustrated by Chen and Zhou ([14], Figure fourteen A) in that the posterior region is preserved in dorso-ventral aspect, judging from the laterally disposed intersegmental membranes. The variable preservational aspects and three-dimensionality of this rod-like structure indicate that it is not an external cuticular feature. In the holotype, the part presents the cuticle wall from one side with the impression of the rod (arrow in Figure 1A), while the counterpart has the rod (slightly darker colour) and the other cuticle wall under it (see detail in Figure 2B). Its consistent three-dimensional shape is possibly the result of early diagenetic mineralization, as opposed to the more unlikely explanation of passive sediment-infilling of a narrow, hollow structure [15]. The latter scenario would require sediment to have entered through the mouth, filled the anterior body cavity and overflowed into the tail cavity, resulting in a more irregular width and relief of the rod-like structure in the posterior body region, but this has not been observed in the available specimens of *Nesonektris*; moreover, sediment infill would have a similar colour to the surrounding matrix, which is at odds with the differential colour of the rod-like structure. The complete mineralization of the rod may have occurred a considerable time after burial, as some specimens show intersegmental membranes impressed into the rod as a result of sediment compaction (Figures 3B, 4B).

The frequent ‘disarticulation’ of the axial structure in the posterior region of *Nesonektris* into a series of short discrete units is inconsistent with the architecture and structural integrity of the gut of vetulicolians, but shows striking similarities to the anatomy and initial decay of the notochord in chordates. In cephalochordates, larval urochordates and some larval vertebrates (lampreys), the notochord comprises thin, stacked disc-shaped units enclosed in a sheath [16-18]. The observed notochord blocks (Figures 2A, C, and between the yellow arrows in Figure 2E) would correspond to sets with numerous discs; and smaller sets making up each sub-segmental block (between white arrows in Figure 2E), with no individual discs being recognized with certainty. Taphonomic experiments on chordates [19,20] have shown that the notochord is highly decay-resistant, considerably more than the gut (Sansom et al. [20], Figure eight), with the contained tissue initially condensing and breaking up, often into blocks at regular intervals (arrows in Figure 2F, and Sansom et al. [20], Figures six B and seven C,D). This style of decay could explain the separated and offset sections of the axial structure in several specimens of *Nesonektris* (Figure 2), possibly as a result of body contortion (Figure 1A-C, F) and subsequent compaction during burial. This is supported by the fact that distorted, yet complete specimens of *Nesonektris* are commonly preserved in siltstones – often associated with bent, fully articulated trilobite exoskeletons – that have been rapidly deposited by sediment gravity flows [21]. The blocks that are considerably displaced (Figure 2A, D) can only be explained in the context of a notochord if the sheath had partially decayed and/or ruptured.

The interpretation of the axial structure as a notochord fits the putative functional morphology of the posterior region in vetulicolians. In modern chordates, the notochord is a major structural support and plays a role in locomotion by stiffening the body and assisting the antagonistic action of the muscles to generate lateral propulsive movements. In pelagic tunicates (including ascidian larvae), the notochord, which plays a role in their undulatory swimming, is restricted to, and occupies a large area of the tail region [22,23], proportionately similar to *Nesonektris* and in the same dorso-ventral location of the tail. The large relative size of the rod of *Nesonektris* did not affect the physical ability to bend smoothly during swimming, because the folded tail in specimens like the one in Figure 1A-C indicates that flexibility was not compromised. The volume of the rod provided ample room for muscles, gut and other internal organs. Recent evidence [7,8] favours the flanges in the posterior region of vetulicolians being vertical and the tail flexing laterally (rather than dorso-ventrally). The intersegmental membranes – also present in *Nesonektris* (Figures 1D, G, 4) – are bilaterally disposed and narrow towards the dorsal and ventral midline, and there is a dorso-ventral asymmetry in the most proximal segments of the tail due to the forward-slanted articulation with the antero-dorsal region (e.g., Aldridge et al. [6], Plate two, one and seven; Shu et al. [7], Figure one; Ou et al. [9], Figure seven A,B).

Counterarguments can be raised to the interpretation of the rod-like structure as a notochord, some based on inconsistencies in structure or inferred function, some based on correspondences to a gut or body cavity, and others taphonomic. These include the following:

1. The rod-like structure of *Nesonektris* has a considerable diameter, being larger than the notochord in modern adult chordates. This could raise questions about its physical properties, e.g. whether it was too bulky to bend and recoil efficiently during swimming. Nevertheless, the rod-like structure also seems to be disproportionately large to be a gut, as it is wider than the ventral food gutter and considerably more so than the gut in other vetulicolians (e.g. Aldridge et al. [6], *Vetulicola* sp. in Plate one, Figure seven, and *Pomatrum* in Plate four, Figure eight, twelve; or Ou et al. [9], Figure six F, H, I);
2. Guts are common in the Cambrian fossil record, in part because they are prone to early diagenetic mineralization, whereas well-corroborated notochords have a depauperate Cambrian record. Although originally recognized in yunnanozoans as a thin anterior notochord, considerably thickening backwards (e.g. Mallatt and Chen [24], figures One A and Ten), later studies question this interpretation [25]. *Pikaia* was originally described as having a thick dorsal notochord, but this is now regarded as a ‘dorsal organ’, while the notochord is proposed to be very thin and in an axial position deep inside the body (see Conway Morris and Caron [26] and discussion in Mallat and Holland [11]). The latest study on *Metaspriggina* ([27], Figures One a–d and Extended Data figure One a–d, f) identifies a thin (0.25 mm in diameter), elongate strand lying on the midline, opposite the zone of myotomal closure as a notochord. In the early fish *Myllokunmingia*, Shu et al. [28] describe a thin but fairly prominent strand ([28], figures Two a and Three) as the probable incomplete remains of the notochord. Thus, most of the latest descriptions of Cambrian chordates only refer to thin notochords, similar in position but different in width to the axial rod of *Nesonektris*;
3. The rod-like structure ends terminally (like a gut) at a depression (like an anus), whereas notochords are usually more dorsally located and extend out into a post-anal tail (see discussion below) that tapers at the end.

Another interpretation for the rod (besides a notochord or a gut) is a coelomic cavity, as has been interpreted for a similarly positioned rod-like structure in *Yunnanozoon* [25]. Deuterostome affinities would imply a coelomic cavity that included the gut and other internal organs, such as gonads, and the considerable width of the rod-like structure is consistent with this. We are sceptical about it being a coelomic cavity because it is not feasible that a coelom would fragment into blocks or discs. Rather, it would be expected that a thin bounding membrane (the mesentery) would instead have ruptured, leaving ragged edges, and spilled its content (as would a gut). However, the ‘dorsal organ’ of *Pikaia* appears irregularly ruptured in a few specimens (Conway Morris and Caron [26], figure Five E-G], which has been later suggested as a hydrostatic support system consisting of coelomic chambers with sturdy walls [29].

All vetulicolians have a terminal anus, as is likewise inferred to be the case for *Nesonektris*, which contrasts with the post-anal tail of chordates. However, tunicate larvae have a strip of endoderm in their swimming trunk, a gut remnant that extends to the caudal end. This suggests that no post-anal tail existed in ancestral tunicates [18].

Vetulicolians are usually regarded as pelagic and occasionally nektobenthic [6]. In the Emu Bay Shale they are found as contorted bodies in the siltstone “event” beds, and rare in the finer-sediment (“background”) beds, which is consistent with them being active swimmers in the water column and getting caught in the sediment gravity flows. Despite the lack of gill pouches in the *Nesonektris* specimens, which is regarded as preservational, there is evidence of food gutters with mineralized boluses (Figure 3C, D, F). These food gutters fit the interpretation of Ou et al. [9] that vetulicolians were filter feeders. In this setting, the cavity in the anterior region may show sediment infill from the sediment flows (as is often the case in the larger vetulicolians and in some *Nesonektris* specimens: Figures 1A-C, 3D), but this would not have been actively passed to the gut in the posterior region (as discussed above).

### Phylogenetic analysis

**Figure 5:**
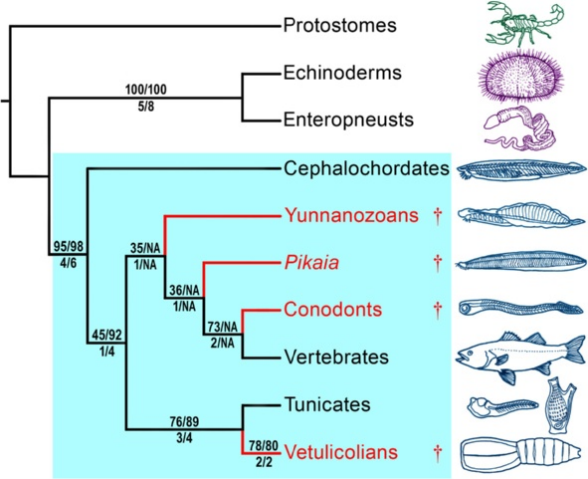
**Phylogenetic position of vetulicolians within Deuterostomia.** Maximum parsimony analysis strict consensus of 65 cladograms (vetulicolians collapsed into a single terminal), based on character matrix in Additional file 3. Blue box encapsulates phylum Chordata; daggers and red branches represent extinct taxa known only from fossils. Parsimony-bootstrap values (above branches) and Bremer support (below) are shown for analyses including/excluding the non-vetulicolian fossil taxa.

**Figure 6:**
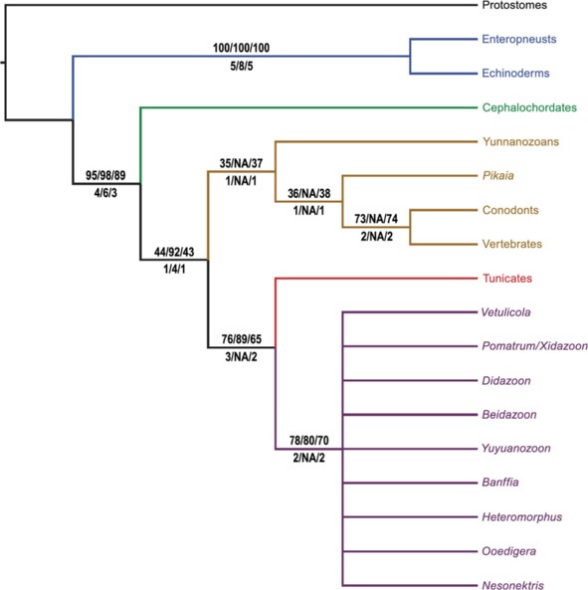
**Strict consensus of 65 cladograms, including all vetulicolian taxa.** Parsimony-bootstrap values (above branches) and Bremer support (below) are shown for analyses as X/Y/Z: X = including non-vetulicolian fossil taxa; Y = excluding the latter taxa; Z = coding the notochord as ‘?’ in *Nesonektris* (char. 12, 27 and 28). All trees 58 steps long.

In our phylogenetic analysis based on maximum parsimony (Figure 5; Methods, Additional files 3–4), taxon sampling, characters and coding largely follow those of Mallatt and Holland [11]. *Yunnanozoon* has been subject to extreme differences in interpretation of its morphological structures depending on whether it is thought to be allied to vertebrates [11] or to non-deuterostome bilaterians [25]. Repeating the analysis with *Yunnanozoon* excluded did not affect tree topology, and clade support also remained similar. The analysis was carried out with PAUP* [30] on the new matrix (17 deuterostome taxa and 33 characters) with a molecular scaffold constraining relationships between living taxa to preserve monophyly of Ambulacraria (i.e. echinoderms and enteropneusts) and of Olfactores (tunicates and vertebrates), which are robustly supported by phylogenomic data [31]. Our results show that vetulicolians form a monophyletic group (Figure 6), rejecting the alternative hypothesis that they are a paraphyletic assemblage at the base of some other deuterostome group [8]. Vetulicolia is resolved as sister group to the tunicates based on the shared presence of a distinctly bipartite body, a stiff cuticle, terminal (rather than ventral) mouth, and a notochord restricted to the posterior region. This hypothesis of a vetulicolian-tunicate clade would be consistent with the cuticle of vetulicolians being homologous with a tunic (char. 6). Also supporting this hypothesis is the observed vestigial gut in the tail of larval tunicates and the dorsal segmentation in the early Cambrian stem-tunicate *Shankouclava* [32]. More broadly, vetulicolians are united with all other chordates based on the presence of a notochord, segmental muscles [11], and a fusiform body in at least some part of their life cycle. As such, vetulicolians belong to both crown-group Chordata [22] and crown-group Olfactores (Vertebrata + Urochordata) (Figures 5, 6). Inclusion of *Nesonektris* in the matrix (and thus the presence of a notochord in a vetulicolian) is not critical for resolution of vetulicolians as chordates: the same topology is retrieved without *Nesonektris*. However, inclusion of this new taxon and coding the presence of a notochord increases support for most clades, e.g. bootstrap support increases by 13%, 5% and 6% for Vetulicolia + Urochordata, Olfactores, and Chordata, respectively, and Bremer support for these nodes increases by 1 or 2. Although tunicate affinities for vetulicolians have previously been considered [6] or endorsed [5], we stress that this is not an inevitable result of coding vetulicolians as deuterostomes because several other suggested placements within deuterostomes could have been retrieved (e.g., as stem-group Deuterostomia or stem-group Chordata).

Unfortunately, the tree shows no resolution inside the vetulicolians (Figure 6). However, some characters may be homologous amongst some taxa and thus suggest particular clades within vetulicolians. Most specimens of *Nesonektris* are found flat on their sides (Figures 1E,D, 4C), while Figure 1F shows a specimen that is partially rotated along the axis. A partial “twist” has been described in the body of *Heteromorphus* [e.g., Chen [33], figure Five hundred and seven] and a well-developed torsion is found in *Banffia* [13], whose posterior region also ends in a notch (as in *Nesonektris*), but has numerous (>40) segments. *Banffia*’s cylindrical, anterior region has two “narrow, oblique grooves”, which we consider homologous to the lateral grooves in other vetulicolians, and it also lacks evidence for lateral pouches. A subset of the shortest cladograms positions *Nesonektris* as an intermediate form between *Vetulicola*-type (laterally compressed bodies with gill pouches, thickened wall and hepta-segmented posterior region) and *Banffia*-type morphologies.

In the above analysis, *Nesonektris* was scored with the notochord characters; other vetulicolians were conservatively treated as unknown for these notochord traits. Repeating the analysis with *Nesonektris* (and thus all vetulicolians) scored as unknown for notochord characters (12, 27, 28) yields the same tree topology, albeit with a slight drop in support values for the two relevant nodes (tunicates + vetulicolians = Bremer 2/bootstrap 65; Olfactores 1/43, crown chordates 3/89). Thus, the interpretation of vetulicolians as tunicate relatives (and thus crown chordates) does not hinge on the assumption that they possess a notochord. Rather, this interpretation is supported independently by the derived characters they share with urochordates, and the high position of urochordates within chordates revealed by phylogenomic data. Characters that optimise as apomorphic for a tunicate-vetulicolian clade (between node 31 and 27 in Additional file 4) are: distinct anterior and posterior body regions (char. 1); a thick cuticle (char. 6); a non-fusiform body, at least in larval stages (char. 8); terminal position of the mouth (char. 16); absence of pharyngeal bars (char. 19); and a notochord restricted to the posterior region of the body (char. 27).

## Conclusions

The phylogenetic affinity between vetulicolians and tunicates has significant implications for the ancestral morphology of the Urochordata (tunicates), which has been a continuing conundrum in chordate phylogeny [17]. The Olfactores concept suggests that successive living sister taxa to tunicates are vertebrates and cephalochordates [34,35], thus indicating that tunicates are derived from a free-swimming, segmented, amphioxus-like animal with a notochord, neural crest cells (or homologous precursor-cell populations), neural tube, striated heart muscle and segmented lateral muscles, but lacking an atrium [17,31,34,36]. The dorsal segmentation of the tail in the Cambrian *Shankouclava* [32] points to actively-moving, ascidian-like early tunicates. The common ancestor to the vetulicolian + tunicate clade is inferred to have been a pelagic animal with a thick cuticle for protection, a large filter-feeding pharynx in the anterior body region, and a segmented, propelling posterior region with a notochord and a terminal anus. If the free-swimming appendicularians are considered basal to all other tunicates [31], the most parsimonious scenario implies that the common ancestor of tunicates also retained all these traits; the benthic sessile adult morphology in many tunicates (e.g., ascidians) optimises as a derived specialisation [31,34].

The vetulicolians could have filled the ecological niche of medium-large suspension feeders in the Cambrian, supporting the idea that chordates arose as a clade of suspension feeders (as is the case for early vertebrates such as *Haikouichthys* as well as for yunnanozoans and modern amphioxus, tunicates, and larval lampreys), rather than as predators. They would have been blind and slow moving, but relying on their size and tough cuticle for protection. The interpretation of vetulicolian morphology has been fraught with problems [4,6], but mounting evidence, notably the presence of pharyngeal gill slits [9], indicates that they belong to the deuterostome total group [5,10]. Evidence for a possible notochord in vetulicolians provides new support for placing this abundant and diverse Cambrian group more crownward and, as a consequence, substantially increases the diversity, disparity and abundance of Cambrian chordates [26-28,31,32,37].

## Methods

### Material

The material (Additional file 2) is housed in the palaeontological collections of the South Australian Museum (Adelaide). Preparation used a compressed-air micro-jack to reveal features hidden by overlying matrix. Photographs were taken with a Canon EOS 5D digital SLR camera with a Canon MP-E 65 mm 1–5× macro lens, usually with low angle light to enhance the relief of the fossil. Figure 1C is a composite of the camera lucida drawings of the holotype made with an Olympus SZ10 binocular microscope. Figures were assembled with Adobe Photoshop CS3.

### Character description

1. Distinct anterior and posterior body. 0 = absent; 1 = present.
2. Segmentation or metamerism. 0 = absent; 1 = present.
3. Muscle segments. 0 = absent; 1 = present.
4. Myomeres. 0 = absent; 1 = present.
5. Myomere shape. 0 = more or less vertical; 1 = present and sharply angled. Since this character is only applicable to taxa with myomeres (char. 4), we have coded it as inapplicable in protostomes and echinoderms.
6. Thick cuticle. 0 = absent; 1 = present. Mallatt and Holland [11] coded tunicates as lacking a cuticle due to it containing cellulose from a bacterial gene transfer origin. We here code all taxa with a thick cuticle with state 1, without regard for whether the cuticle contains cellulose or not (since this condition is usually indeterminate in fossil taxa).
7. Body shape, laterally compressed and fusiform. 0 = absent; 1 = present.
8. Whole body fusiform. 0 = no; 1 = yes. Since this character is only applicable to taxa with fusiform bodies (char. 7), we have coded it as inapplicable in protostomes, echinoderms and enteropneusts. Vetulicolians and tunicates (at least in part of their life cycle) have a wide, non-fusiform anterior region of the body.
9. Dorsal and/or ventral fin or keel. 0 = absent; 1 = present.
10. Fin rays. 0 = absent; 1 = present. Since this character is only applicable to taxa with fins or keels (char. 9), we have coded it as inapplicable in protostomes, echinoderms and enteropneusts, plus those vetulicolians without keels (*Didazoon* and *Yuyuanozoon*).
11. Position of anus. 0 = terminal; 1 = post-anal tail (sub-terminal anus). We have coded this character with both terminal and subterminal for enteropneusts rather than as ‘?’ as in [11].
12. Notochord. 0 = absent; 1 = present.
13. Paired eyes. 0 = absent; 1 = present. We have opted for a cautious approach and coded the presence of eyes in yunnanozoans as possible (‘?’) rather than demonstrably ‘present’ [11] or ‘absent’ [25].
14. Dorsal nerve cord. 0 = absent; 1 = present.
15. Median ventral, or dorsal and ventral, longitudinal blood vessels running most of the body length. 0 = present; 1 = absent.
16. Position of mouth. 0 = terminal; 1 = ventral, subterminal.
17. Buccal cavity. 0 = absent; 1 = present. We have coded this character in yunnanozoans as uncertain (‘?’), given differing recent views e.g. ‘present’ [11] or ‘absent’ [26]. We have coded this character as present for conodonts [38].
18. Wide pharyngeal cavity or wide anterior foregut. 0 = absent; 1 = present.
19. Pharyngeal bars. 0 = absent; 1 = present.
20. Gills. 0 = absent; 1 = present and internal; 2 = present and external.
21. Number of branchial units or bars in the pharynx. 0 = none; 1 = intermediate (5–9); 2 = numerous (10 or more).
22. Pharyngeal slits or pores. 0 = absent; 1 = present.
23. Atrium. 0 = absent; 1 = present.
24. Suspension feeder. 0 = absent; 1 = bona fide suspension feeder; 2 = atypical, facultative suspension feeder or deposit feeder that traps many particles on external mucus. Suspension feeding as well as predation has been considered as the ancestral mode in vertebrates [39], so we have coded this character as both ‘absent’ (0) and ‘present’ (1).
25. Chordate pattern of cilia tracts in pharynx. 0 = absent; 1 = limited, mostly to pharyngeal arches only; 2 = complete.
26. Pre-oral skirt, hood, or bilobed head (e.g., as seen in *Pikaia*). 0 = absent; 1 = present.
27. Notochord extension. 0 = extends along most of body; 1 = restricted to posterior region of body. Although not included in Mallatt and Holland [11], this character is phylogenetically informative within taxa that have a notochord (char. 12), and is otherwise inapplicable. We have coded as unknown for vetulicolians other than *Nesonektris*, pending a revision of those fossils.
28. Notochord type. 0 = “stack of coins”; 1 = vacuolar and longitudinally continuous. This character is only applicable to taxa with a notochord (char. 12). The notochord of cephalochordates and larval urochordates resembles a “stack of coins” [40,41], whereas it is vacuolar and longitudinally continuous in adult vertebrates.
29. Orifice to the pharyngeal cavity. 0 = simple; 1 = surrounded by cuticular lip; 2 = surrounded by double circlet of plates. This character is only applicable to vetulicolians.
30. Lateral groove. 0 = absent; 1 = present. This character is only applicable to vetulicolians, and is redefined from Aldridge et al. [6] (char. 32 therein). We have coded ‘?’ for *Banffia* because the anterior body is divided into two “carapaces” separated by two “narrow, oblique grooves” (Caron [13], Page ninety-nine); which we consider homologous to the straight lateral groove of the rest of vetulicolians. These grooves are often labelled as carapace “margins” in Caron’s [13] figures. In *Yuyuanozoon* we have coded this character as 0 because the groove does not extend the whole length of the anterior body (e.g. Aldridge et al. [6], Plate five, Figures three and five), as it does in all other vetulicolians.
31. Shape of anterior body region. 0 = ovoid; 1 = subquadrate. This character is only applicable to vetulicolians, and is from Aldridge et al. [6] (char. 35 therein).
32. Shape of terminal segment. 0 = not notched; 1 = notched. This character is only applicable to vetulicolians. The end of the posterior body region is generally rounded in most vetulicolians, with the exception of *Banffia* (Caron [13], Figures four E, eighteen G-I) and *Nesonektris* (Figures 1A-E,G, 2B-D), which have a caudal notch. This character is redefined and the states inverted from Aldridge et al. [6] (char. 36 therein).
33. Number of segments in posterior region. 0 = seven segments, 1 = twenty or more segments. This character is only applicable to vetulicolians, and no taxa have any other states. *Pomatrum* (Aldridge et al. [6], Page one hundred and fortyseven), *Heteromorphus* (Aldridge et al. [6], Page one hundred and fifty) and *Banffia* [13] all have 20 or more segments. This character is redefined from Aldridge et al. [6] (char. 37 therein).

### Explanation of character matrix

Characters 1–26 are formulated and, except where noted above, coded as in the matrix by Mallatt and Holland [11]. We excluded *Herpetogaster* because it has little bearing on interrelationships within Chordata [11], but included echinoderms due to their importance in deuterostome evolution, and scored all described vetulicolian genera, including *Nesonektris*. The vetulicolian taxa *Pomatrum* and *Xidazoon* are considered as a single terminal – as have been *Beidazoon* and *Bullivetula* – notwithstanding some on-going discussion relating to their synonymy [6,7,9]. We have added new characters (27–33) relevant to notochord structure (27, 28) and vetulicolian ingroup relationships (29–33). Differences in coding between our matrix and ref. 11 are discussed above; in particular, some protostome characters (2, 3, 6, 13, 16, 20 and 24) coded by them as unknown (?) are here considered known, but polymorphic/variable (0&1). Other characters can only be scored when another character is present (e.g., notochord type can only be scored when a notochord is present); we use inapplicable (“-”) when they cannot be scored.

The first quantitative phylogenetic analysis of vetulicolians [6] treated their membership in either Protostomia or Deuterostomia to be an open question and accordingly sampled numerous protostome representatives in the ingroup to test affinities to kinorhynchs or arthropods. Subsequent description of pharyngeal structures in particular have strengthened the case for deuterostome affinities for vetulicolians [8-10] and accordingly our analysis is designed with a single protostome outgroup to root the Deuterostomia.

All parsimony analyses used PAUP* v4.06 [30], with heuristic searches employing 100 random stepwise additional sequences and bootstrapping employing 1000 replicates. Analyses enforced a molecular scaffold for extant taxa that is robustly supported by phylogenomic data [31,35] and contrasts with former morphological hypotheses [40,42]; i.e. (Protostomes, ((Echinoderms, Enteropneusts), (Cephalochordates, (Tunicates, Vertebrates)))).

Analyses were performed with all taxa included, as well as with only ‘extant plus vetulicolian’ taxa. Both analyses resulted in identical trees (for taxa in common). The full matrix, PAUP* search commands, and all (65) most-parsimonious trees found in the all-taxon analysis, are presented in Nexus format in Additional file 3. Bootstrap and Bremer support values for all clades found in both analyses are shown in Figures 5, 6.

Analyses without a molecular scaffold also resulted in vetulicolians falling within Chordata, again as sister group of tunicates. Because of the relatively few informative characters (33) relative to the number of potential ingroup taxa (11), without a molecular backbone there was good resolution only in the analyses when just extant taxa and vetulicolians were included. In the strict consensus tree of this analysis, the tunicate-vetulicolian clade formed a trichotomy with cephalochordates and vertebrates.

## Additional files

Additional file 1:
**Taxonomic list of vetulicolians described to date.**
Additional file 2:
**Referred material.**
Additional file 3:
**Character matrix in nexus format, with PAUP commands.**
Additional file 4:
**Apomorphies in majority-rule consensus of the 65 most parsimonious trees.**
