# Supplementary material for: A new vetulicolian from Australia and its bearing on the chordate affinities of an enigmatic Cambrian group
Source: BMC Evol Biol. 2014 Oct 21;14:214. doi: 10.1186/s12862-014-0214-z (PMC4203957; doi:10.1186/s12862-014-0214-z)
Supplement: Additional file 1: — Taxonomic list of vetulicolians described to date. [file 12862_2014_214_MOESM1_ESM.doc]

**Additional file 1: Taxonomic list of vetulicolians described to date** (after Aldridge et al. [6] and Vinther et al. [8]).

Class Vetulicolida Chen & Zhou 1997 [14]

Order Vetulicolata (Hou & Bergström 1997) [2]

Family Vetulicolidae Hou & Bergström 1997 [2]

Genus *Vetulicola* Hou 1987 [1]

**·** *V. cuneata* Hou 1987 [1]

**·** *V. rectangulata* Luo & Hu *in* Luo *et al*. 1999

**·** *V. monile* Aldridge et al. 2007 [6]

**·** *V. gangtoucunensis* Luo, Fu & Hu *in* Luo *et al*. 2005

**·** *V. longbaoshanensis* Yang, Hou, Cong *et al*. 2010 [12]

**·** *V.* sp. Aldridge *et al*. 2007 [6]

Genus *Yuyuanozoon* Chen, Feng & Zhu *in* Chen *et al*. 2003

**·** *Y. magnificissimi* Chen, Feng & Zhu *in* Chen *et al*. 2003

Genus *Ooedigeria* Vinther, Smith & Harper 2011 [8]

**·** *O. peeli* Vinther, Smith & Harper 2011 [8]

Family Beidazoonidae Chen & Zhou 1997 [14]

Genus *Beidazoon* Shu 2005 (=*Bullivetula* Aldridge et al. 2007)

**·** *B. venustum* Shu 2005 (=*B. variola* Aldridge et al. 2007)

Family Didazoonidae Shu & Han *in* Shu *et al*. 2001 [3]

Genus *Didazoon* Shu & Han *in* Shu *et al*. 2001 [3]

**·** *D. hoae* Shu & Han *in* Shu *et al*. 2001 [3]

**·** *D.?* sp. Aldridge *et al*. 2007 [6]

Genus *Pomatrum* Luo & Hu *in* Luo *et al*. 1999

**·** *P. ventralis* Luo & Hu *in* Luo *et al*. 1999

(Genus *Xidazoon* Shu, Conway Morris & Zhang 1999 *in* Shu *et al*., 1999

**·** *X. stephanus* Shu, Conway Morris & Zhang 1999 *in* Shu *et al*., 1999)

Order Banffiata Aldridge et al., 2007 [6]

Family Banffidae Caron 2006 [13]

Genus *Heteromorphus* Luo & Hu *in* Luo *et al*. 1999

**·** *H. confusus* (Chen & Zhou 1997) [14]

**·** *H. longicaudatus* Luo & Hu *in* Luo *et al*. 1999

Genus *Banffia* Walcott 1911

**·** *B. constricta* Walcott 1911

**References** (not cited in main text)

Luo H, Hu S, Chen L, Zhang, S, Tao Y: *Early Cambrian Chengjiang fauna from Kunming Region, China*. Kunming: Yunnan Science and Technology Press; 1999.

Luo H, Fu X, Hu X, Li Y, Chen L, You T, Liu Q: **New vetulicoliids from the Lower Cambrian Guanshan Fauna, Kunming**. *Acta Geo Sinica* 2005*,* **79**:1–6.

Chen A, Feng H, Zhu M, Ma D, Li M: **A new vetulicolian from the Early Cambrian Chengjiang fauna in Yunnan of China**. *Acta Geol Sinica* 2003, **77**:281–287.

Shu D: **On the phylum Vetulicolia**. *Chinese Sci Bull* 2005, **50**, 2342–2354.

Shu D, Conway Morris S, Zhang XL, Chen L, Li Y, Han J: **A pipiscid-like fossil from the Lower Cambrian of south China**. *Nature* 1999, **400**:746–749.

Walcott CD: **Middle Cambrian annelids. Cambrian Geology and Paleontology II**. *Smith Misc Coll* 1911, **57**:109–144.
