# Supplementary material for: A new vetulicolian from Australia and its bearing on the chordate affinities of an enigmatic Cambrian group
Source: BMC Evol Biol. 2014 Oct 21;14:214. doi: 10.1186/s12862-014-0214-z (PMC4203957; doi:10.1186/s12862-014-0214-z)
Supplement: Additional file 2: — Referred material. [file 12862_2014_214_MOESM2_ESM.doc]

**Additional file 2: Referred material.** All Emu Bay Shale material was collected from Buck Quarry, with the exception of two non-figured specimens from the shoreline locality [20], and is housed in the collections of the South Australian Museum, Adelaide. The Emu Bay Shale Konservat-Lagerstätte is found in Big Gully, northwest of Kingscote, Kangaroo Island (South Australia) and is early Cambrian (Cambrian Series 2, Stage 4, Botoman equivalent) in age.

Catalogue Numbers

Holotype: SAM P45212a,b

Paratypes (figured): SAM P45215a,b

SAM P46336a,b

SAM P47152a,b

SAM P47166a,b

SAM P47168a,b

SAM P48013a,b

SAM P48015a,b

SAM P48073a,b

SAM P48093a,b

SAM P48105a,b

SAM P49076a,b

SAM P49080a,b

SAM P49084

SAM P49147a,b

SAM P49662a,b

Other (non-figured): About 150 specimens in the collections of the South Australian Museum.
