# Supplementary material for: A new vetulicolian from Australia and its bearing on the chordate affinities of an enigmatic Cambrian group
Source: BMC Evol Biol. 2014 Oct 21;14:214. doi: 10.1186/s12862-014-0214-z (PMC4203957; doi:10.1186/s12862-014-0214-z)
Supplement: Additional file 3: — Character matrix in nexus format, with PAUP commands. [file 12862_2014_214_MOESM3_ESM.doc]

**Additional file 3: Character matrix in nexus format, with PAUP commands.**

#NEXUS

BEGIN TAXA;

TITLE Taxa;

DIMENSIONS NTAX=18;

TAXLABELS

Protostomes

Echinoderms

Enteropneusts

Vetulicola

Pomatrum_Xidazoon

Didazoon

Beidazoon

Yuyuanozoon

Banffia

Heteromorphus

Ooedigera

Nesonektris

Tunicates

Cephalochordates

Pikaia

Yunnanozoans

Vertebrates

Conodonts;

END;

BEGIN CHARACTERS;

DIMENSIONS NCHAR=33;

FORMAT SYMBOLS= " 0 1 2" MISSING=? GAP=- ;

MATRIX

[ 10 20 30 ]

[ . . . ]

Protostomes 0(01)(01)0-(01)0-0-00(01)00(01)-00(01)--0(01)00-------

Echinoderms 00?0-00-0-10000?-000--0100-------

Enteropneusts 00???00-0-(01)0000?0110210210-------

Vetulicola 11110110100?0??001??110??0??11000

Pomatrum_Xidazoon 11110110100?0??001??110??0??21001

Didazoon 111101100-0?0??001??110??0??21000

Beidazoon 11110110100?0??001??110??0??11100

Yuyuanozoon 111101100-0?0??001??110??0??00000

Banffia 11110110000?0??001??1?0??0??2?011

Heteromorphus 11110110100?0??001??1?0??0???1001

Ooedigera 11110110100?0??001??110??0??11000

Nesonektris 1111011010010??001????0??01011110

Tunicates 1????11010110110110021112010-----

Cephalochordates 0111101110110111111021112000-----

Pikaia 0111001110??0?01?1?21100??0?-----

Yunnanozoans 011100111011?1?1?1111111?10?-----

Vertebrates 01111011111111111111110(01)2101-----

Conodonts 0111101111?11??111??1??00???-----

;

END;

[Unbracket commands below to run search under molecular backbone constraints]

[

BEGIN PAUP;

SET MAXTREES = 200000 tcompress = yes torder = left showtaxnum = no taxlabels = full;

SET storetreewts = yes;

SET CRITERION = parsimony;

PSET mstaxa = variable;

LOG start file = MP_output.txt replace = yes;

CONSTRAINTS molecbackbone (backbone) = [&R] (((Echinoderms,Enteropneusts),(Cephalochordates,(Tunicates,Vertebrates))),Protostomes);

OUTGROUP Protostomes;

SET ROOT = OUTGROUP OUTROOT = monophyl;

HSEARCH addseq = random nreps = 1000 rstatus = yes nchuck = 1000 chuckscore = 1 enforce = yes constraints = molecbackbone [keep=58];

SAVETREES File = VetulicolansOnly_all_parsimonious.trees brlens = yes root = yes replace = yes;

CONTREE / treefile = VetulicolansAll_con.tree replace = yes;

LOG stop;

END;

]

[All 65 Most-Parsimonious trees for full taxon set, Length = 58; remove brackets below to load trees]

[

Begin trees;

Translate

1 Protostomes,

2 Echinoderms,

3 Enteropneusts,

4 Vetulicola,

5 Pomatrum_Xidazoon,

6 Didazoon,

7 Beidazoon,

8 Yuyuanozoon,

9 Banffia,

10 Heteromorphus,

11 Ooedigera,

12 Nesonektris,

13 Tunicates,

14 Cephalochordates,

15 Pikaia,

16 Yunnanozoans,

17 Vertebrates,

18 Conodonts

;

tree PAUP_1 = [&R] (1:0,((2:2,3:2):0,(((((4:0,(7:0,12:1):1,11:0):1,(5:0,10:0):1,(6:0,8:2,9:2):1):3,13:1):6,((15:2,(17:0,18:1):3):2,16:0):3):1,14:1):10):3);

tree PAUP_2 = [&R] (1:0,((2:2,3:2):0,((((((4:0,(7:0,12:1):1,11:0):1,(6:0,8:2,9:2):1):1,5:0,10:0):3,13:1):6,((15:2,(17:0,18:1):3):2,16:0):3):1,14:1):10):3);

tree PAUP_3 = [&R] (1:0,((2:2,3:2):0,((((4:0,((5:0,10:0):1,(6:0,8:2,9:2):1):1,(7:0,12:1):1,11:0):3,13:1):6,((15:2,(17:0,18:1):3):2,16:0):3):1,14:1):10):3);

tree PAUP_4 = [&R] (1:0,((2:2,3:2):0,(((((4:0,((5:0,10:0):1,(6:0,8:2,9:2):1):1,11:0):0,(7:0,12:1):1):3,13:1):6,((15:2,(17:0,18:1):3):2,16:0):3):1,14:1):10):3);

tree PAUP_5 = [&R] (1:0,((2:2,3:2):0,((((((4:0,((5:0,10:0):1,(6:0,8:2,9:2):1):1,11:0):1,7:0):0,12:1):3,13:1):6,((15:2,(17:0,18:1):3):2,16:0):3):1,14:1):10):3);

tree PAUP_6 = [&R] (1:0,((2:2,3:2):0,(((((4:0,((5:0,10:0):1,(6:0,8:2,9:2):1):1,11:0):1,7:0,12:1):3,13:1):6,((15:2,(17:0,18:1):3):2,16:0):3):1,14:1):10):3);

tree PAUP_7 = [&R] (1:0,((2:2,3:2):0,(((((4:0,(7:0,12:1):1,11:0):0,((5:0,10:0):0,((6:0,8:2):1,9:1):1):2):3,13:1):6,((15:2,(17:0,18:1):3):2,16:0):3):1,14:1):10):3);

tree PAUP_8 = [&R] (1:0,((2:2,3:2):0,((((((4:0,(7:0,12:1):1,11:0):1,((6:0,8:2):0,9:2):1):0,(5:0,10:0):1):3,13:1):6,((15:2,(17:0,18:1):3):2,16:0):3):1,14:1):10):3);

tree PAUP_9 = [&R] (1:0,((2:2,3:2):0,(((((4:0,(7:0,12:1):1,11:0):0,((5:0,10:0):1,(6:0,(8:1,9:2):1):1):1):3,13:1):6,((15:2,(17:0,18:1):3):2,16:0):3):1,14:1):10):3);

tree PAUP_10 = [&R] (1:0,((2:2,3:2):0,((((((4:0,(7:0,12:1):1,11:0):1,(6:0,(8:1,9:2):1):1):0,(5:0,10:0):1):3,13:1):6,((15:2,(17:0,18:1):3):2,16:0):3):1,14:1):10):3);

tree PAUP_11 = [&R] (1:0,((2:2,3:2):0,(((((4:0,(7:0,12:1):1,11:0):0,(5:0,((6:0,8:2):1,9:1):1,10:0):2):3,13:1):6,((15:2,(17:0,18:1):3):2,16:0):3):1,14:1):10):3);

tree PAUP_12 = [&R] (1:0,((2:2,3:2):0,(((((4:0,(7:0,12:1):1,11:0):0,((5:0,((6:0,8:2):1,9:1):1):0,10:0):2):3,13:1):6,((15:2,(17:0,18:1):3):2,16:0):3):1,14:1):10):3);

tree PAUP_13 = [&R] (1:0,((2:2,3:2):0,(((((4:0,(7:0,12:1):1,11:0):0,((5:0,10:0):1,(6:0,8:2,9:2):1):1):3,13:1):6,((15:2,(17:0,18:1):3):2,16:0):3):1,14:1):10):3);

tree PAUP_14 = [&R] (1:0,((2:2,3:2):0,(((((4:0,(7:0,12:1):1,11:0):1,((((5:0,10:0):1,9:1):1,6:0):1,8:1):1):3,13:1):6,((15:2,(17:0,18:1):3):2,16:0):3):1,14:1):10):3);

tree PAUP_15 = [&R] (1:0,((2:2,3:2):0,((((((4:0,(7:0,12:1):1,11:0):1,(6:0,8:2,9:2):1):0,(5:0,10:0):1):3,13:1):6,((15:2,(17:0,18:1):3):2,16:0):3):1,14:1):10):3);

tree PAUP_16 = [&R] (1:0,((2:2,3:2):0,(((((4:0,(7:0,12:1):1,11:0):1,(5:0,10:0):1,(6:0,(8:1,9:2):1):1):3,13:1):6,((15:2,(17:0,18:1):3):2,16:0):3):1,14:1):10):3);

tree PAUP_17 = [&R] (1:0,((2:2,3:2):0,(((((4:0,(7:0,12:1):1,11:0):1,(5:0,9:2,10:0):1,(6:0,8:2):1):3,13:1):6,((15:2,(17:0,18:1):3):2,16:0):3):1,14:1):10):3);

tree PAUP_18 = [&R] (1:0,((2:2,3:2):0,(((((4:0,(7:0,12:1):1,11:0):1,(5:0,10:0):1,((6:0,8:2):0,9:2):1):3,13:1):6,((15:2,(17:0,18:1):3):2,16:0):3):1,14:1):10):3);

tree PAUP_19 = [&R] (1:0,((2:2,3:2):0,((((((4:0,(7:0,12:1):1,11:0):1,((6:0,8:2):0,9:2):1):1,5:0,10:0):3,13:1):6,((15:2,(17:0,18:1):3):2,16:0):3):1,14:1):10):3);

tree PAUP_20 = [&R] (1:0,((2:2,3:2):0,((((4:0,((5:0,10:0):0,((6:0,8:2):1,9:1):1):2,(7:0,12:1):1,11:0):3,13:1):6,((15:2,(17:0,18:1):3):2,16:0):3):1,14:1):10):3);

tree PAUP_21 = [&R] (1:0,((2:2,3:2):0,(((((4:0,((5:0,10:0):0,((6:0,8:2):1,9:1):1):2,11:0):0,(7:0,12:1):1):3,13:1):6,((15:2,(17:0,18:1):3):2,16:0):3):1,14:1):10):3);

tree PAUP_22 = [&R] (1:0,((2:2,3:2):0,(((((4:0,(7:0,12:1):1,11:0):2,5:0,((6:0,8:2):1,9:1):1,10:0):3,13:1):6,((15:2,(17:0,18:1):3):2,16:0):3):1,14:1):10):3);

tree PAUP_23 = [&R] (1:0,((2:2,3:2):0,((((((4:0,(7:0,12:1):1,11:0):1,10:0):1,5:0,((6:0,8:2):1,9:1):1):3,13:1):6,((15:2,(17:0,18:1):3):2,16:0):3):1,14:1):10):3);

tree PAUP_24 = [&R] (1:0,((2:2,3:2):0,(((((((4:0,(7:0,12:1):1,11:0):2,(6:0,8:2):0):1,9:1):1,5:0,10:0):3,13:1):6,((15:2,(17:0,18:1):3):2,16:0):3):1,14:1):10):3);

tree PAUP_25 = [&R] (1:0,((2:2,3:2):0,(((((4:0,(7:0,12:1):1,11:0):1,(5:0,((6:0,8:2):1,9:1):1):1,10:0):3,13:1):6,((15:2,(17:0,18:1):3):2,16:0):3):1,14:1):10):3);

tree PAUP_26 = [&R] (1:0,((2:2,3:2):0,((((((4:0,(7:0,12:1):1,11:0):1,10:0):0,(5:0,((6:0,8:2):1,9:1):1):1):3,13:1):6,((15:2,(17:0,18:1):3):2,16:0):3):1,14:1):10):3);

tree PAUP_27 = [&R] (1:0,((2:2,3:2):0,((((((((4:0,(7:0,12:1):1,11:0):2,8:1):1,6:0):1,9:1):1,5:0,10:0):3,13:1):6,((15:2,(17:0,18:1):3):2,16:0):3):1,14:1):10):3);

tree PAUP_28 = [&R] (1:0,((2:2,3:2):0,(((((((4:0,(7:0,12:1):1,11:0):2,6:0,8:2):1,9:1):1,5:0,10:0):3,13:1):6,((15:2,(17:0,18:1):3):2,16:0):3):1,14:1):10):3);

tree PAUP_29 = [&R] (1:0,((2:2,3:2):0,((((((4:0,(7:0,12:1):1,11:0):1,(6:0,8:2):1):1,5:0,9:2,10:0):3,13:1):6,((15:2,(17:0,18:1):3):2,16:0):3):1,14:1):10):3);

tree PAUP_30 = [&R] (1:0,((2:2,3:2):0,(((((((4:0,(7:0,12:1):1,11:0):1,(6:0,8:2):1):1,5:0,10:0):0,9:2):3,13:1):6,((15:2,(17:0,18:1):3):2,16:0):3):1,14:1):10):3);

tree PAUP_31 = [&R] (1:0,((2:2,3:2):0,((((((4:0,(7:0,12:1):1,11:0):1,(6:0,(8:1,9:2):1):1):1,5:0,10:0):3,13:1):6,((15:2,(17:0,18:1):3):2,16:0):3):1,14:1):10):3);

tree PAUP_32 = [&R] (1:0,((2:2,3:2):0,((((((4:0,((5:0,10:0):0,((6:0,8:2):1,9:1):1):2,11:0):1,7:0):0,12:1):3,13:1):6,((15:2,(17:0,18:1):3):2,16:0):3):1,14:1):10):3);

tree PAUP_33 = [&R] (1:0,((2:2,3:2):0,(((((4:0,((5:0,10:0):0,((6:0,8:2):1,9:1):1):2,11:0):1,7:0,12:1):3,13:1):6,((15:2,(17:0,18:1):3):2,16:0):3):1,14:1):10):3);

tree PAUP_34 = [&R] (1:0,((2:2,3:2):0,((((4:0,(5:0,((6:0,8:2):1,9:1):1,10:0):2,(7:0,12:1):1,11:0):3,13:1):6,((15:2,(17:0,18:1):3):2,16:0):3):1,14:1):10):3);

tree PAUP_35 = [&R] (1:0,((2:2,3:2):0,((((4:0,((5:0,((6:0,8:2):1,9:1):1):0,10:0):2,(7:0,12:1):1,11:0):3,13:1):6,((15:2,(17:0,18:1):3):2,16:0):3):1,14:1):10):3);

tree PAUP_36 = [&R] (1:0,((2:2,3:2):0,((((4:0,((((5:0,10:0):1,9:1):1,6:0):1,8:1):2,(7:0,12:1):1,11:0):3,13:1):6,((15:2,(17:0,18:1):3):2,16:0):3):1,14:1):10):3);

tree PAUP_37 = [&R] (1:0,((2:2,3:2):0,((((4:0,((5:0,10:0):1,(6:0,(8:1,9:2):1):1):1,(7:0,12:1):1,11:0):3,13:1):6,((15:2,(17:0,18:1):3):2,16:0):3):1,14:1):10):3);

tree PAUP_38 = [&R] (1:0,((2:2,3:2):0,((((4:0,(((5:0,10:0):1,9:1):1,(6:0,8:2):0):2,(7:0,12:1):1,11:0):3,13:1):6,((15:2,(17:0,18:1):3):2,16:0):3):1,14:1):10):3);

tree PAUP_39 = [&R] (1:0,((2:2,3:2):0,((((4:0,((5:0,9:2,10:0):1,(6:0,8:2):1):1,(7:0,12:1):1,11:0):3,13:1):6,((15:2,(17:0,18:1):3):2,16:0):3):1,14:1):10):3);

tree PAUP_40 = [&R] (1:0,((2:2,3:2):0,(((((4:0,(5:0,((6:0,8:2):1,9:1):1,10:0):2,11:0):0,(7:0,12:1):1):3,13:1):6,((15:2,(17:0,18:1):3):2,16:0):3):1,14:1):10):3);

tree PAUP_41 = [&R] (1:0,((2:2,3:2):0,(((((4:0,((5:0,((6:0,8:2):1,9:1):1):0,10:0):2,11:0):0,(7:0,12:1):1):3,13:1):6,((15:2,(17:0,18:1):3):2,16:0):3):1,14:1):10):3);

tree PAUP_42 = [&R] (1:0,((2:2,3:2):0,(((((4:0,((((5:0,10:0):1,9:1):1,6:0):1,8:1):2,11:0):0,(7:0,12:1):1):3,13:1):6,((15:2,(17:0,18:1):3):2,16:0):3):1,14:1):10):3);

tree PAUP_43 = [&R] (1:0,((2:2,3:2):0,(((((4:0,((5:0,10:0):1,(6:0,(8:1,9:2):1):1):1,11:0):0,(7:0,12:1):1):3,13:1):6,((15:2,(17:0,18:1):3):2,16:0):3):1,14:1):10):3);

tree PAUP_44 = [&R] (1:0,((2:2,3:2):0,(((((4:0,(((5:0,10:0):1,9:1):1,(6:0,8:2):0):2,11:0):0,(7:0,12:1):1):3,13:1):6,((15:2,(17:0,18:1):3):2,16:0):3):1,14:1):10):3);

tree PAUP_45 = [&R] (1:0,((2:2,3:2):0,(((((4:0,((5:0,9:2,10:0):1,(6:0,8:2):1):1,11:0):0,(7:0,12:1):1):3,13:1):6,((15:2,(17:0,18:1):3):2,16:0):3):1,14:1):10):3);

tree PAUP_46 = [&R] (1:0,((2:2,3:2):0,((((((4:0,(((5:0,10:0):1,9:1):1,(6:0,8:2):0):2,11:0):1,7:0):0,12:1):3,13:1):6,((15:2,(17:0,18:1):3):2,16:0):3):1,14:1):10):3);

tree PAUP_47 = [&R] (1:0,((2:2,3:2):0,((((((4:0,((5:0,((6:0,8:2):1,9:1):1):0,10:0):2,11:0):1,7:0):0,12:1):3,13:1):6,((15:2,(17:0,18:1):3):2,16:0):3):1,14:1):10):3);

tree PAUP_48 = [&R] (1:0,((2:2,3:2):0,((((((4:0,(5:0,((6:0,8:2):1,9:1):1,10:0):2,11:0):1,7:0):0,12:1):3,13:1):6,((15:2,(17:0,18:1):3):2,16:0):3):1,14:1):10):3);

tree PAUP_49 = [&R] (1:0,((2:2,3:2):0,((((((4:0,(((5:0,10:0):1,9:1):1,6:0,8:2):2,11:0):1,7:0):0,12:1):3,13:1):6,((15:2,(17:0,18:1):3):2,16:0):3):1,14:1):10):3);

tree PAUP_50 = [&R] (1:0,((2:2,3:2):0,((((((4:0,((((5:0,10:0):1,9:1):1,6:0):1,8:1):2,11:0):1,7:0):0,12:1):3,13:1):6,((15:2,(17:0,18:1):3):2,16:0):3):1,14:1):10):3);

tree PAUP_51 = [&R] (1:0,((2:2,3:2):0,((((((4:0,((5:0,9:2,10:0):1,(6:0,8:2):1):1,11:0):1,7:0):0,12:1):3,13:1):6,((15:2,(17:0,18:1):3):2,16:0):3):1,14:1):10):3);

tree PAUP_52 = [&R] (1:0,((2:2,3:2):0,((((((4:0,((5:0,10:0):1,(6:0,(8:1,9:2):1):1):1,11:0):1,7:0):0,12:1):3,13:1):6,((15:2,(17:0,18:1):3):2,16:0):3):1,14:1):10):3);

tree PAUP_53 = [&R] (1:0,((2:2,3:2):0,(((((4:0,(7:0,12:1):1,11:0):0,(((5:0,10:0):1,9:1):1,6:0,8:2):2):3,13:1):6,((15:2,(17:0,18:1):3):2,16:0):3):1,14:1):10):3);

tree PAUP_54 = [&R] (1:0,((2:2,3:2):0,((((4:0,(((5:0,10:0):1,9:1):1,6:0,8:2):2,(7:0,12:1):1,11:0):3,13:1):6,((15:2,(17:0,18:1):3):2,16:0):3):1,14:1):10):3);

tree PAUP_55 = [&R] (1:0,((2:2,3:2):0,(((((4:0,(((5:0,10:0):1,9:1):1,6:0,8:2):2,11:0):0,(7:0,12:1):1):3,13:1):6,((15:2,(17:0,18:1):3):2,16:0):3):1,14:1):10):3);

tree PAUP_56 = [&R] (1:0,((2:2,3:2):0,(((((4:0,(7:0,12:1):1,11:0):0,(((5:0,10:0):1,9:1):1,(6:0,8:2):0):2):3,13:1):6,((15:2,(17:0,18:1):3):2,16:0):3):1,14:1):10):3);

tree PAUP_57 = [&R] (1:0,((2:2,3:2):0,(((((4:0,(7:0,12:1):1,11:0):0,((5:0,9:2,10:0):1,(6:0,8:2):1):1):3,13:1):6,((15:2,(17:0,18:1):3):2,16:0):3):1,14:1):10):3);

tree PAUP_58 = [&R] (1:0,((2:2,3:2):0,((((((4:0,(7:0,12:1):1,11:0):1,(6:0,8:2):1):0,(5:0,9:2,10:0):1):3,13:1):6,((15:2,(17:0,18:1):3):2,16:0):3):1,14:1):10):3);

tree PAUP_59 = [&R] (1:0,((2:2,3:2):0,(((((4:0,((5:0,10:0):1,(6:0,(8:1,9:2):1):1):1,11:0):1,7:0,12:1):3,13:1):6,((15:2,(17:0,18:1):3):2,16:0):3):1,14:1):10):3);

tree PAUP_60 = [&R] (1:0,((2:2,3:2):0,(((((4:0,((5:0,((6:0,8:2):1,9:1):1):0,10:0):2,11:0):1,7:0,12:1):3,13:1):6,((15:2,(17:0,18:1):3):2,16:0):3):1,14:1):10):3);

tree PAUP_61 = [&R] (1:0,((2:2,3:2):0,(((((4:0,((((5:0,10:0):1,9:1):1,6:0):1,8:1):2,11:0):1,7:0,12:1):3,13:1):6,((15:2,(17:0,18:1):3):2,16:0):3):1,14:1):10):3);

tree PAUP_62 = [&R] (1:0,((2:2,3:2):0,(((((4:0,(((5:0,10:0):1,9:1):1,(6:0,8:2):0):2,11:0):1,7:0,12:1):3,13:1):6,((15:2,(17:0,18:1):3):2,16:0):3):1,14:1):10):3);

tree PAUP_63 = [&R] (1:0,((2:2,3:2):0,(((((4:0,(5:0,((6:0,8:2):1,9:1):1,10:0):2,11:0):1,7:0,12:1):3,13:1):6,((15:2,(17:0,18:1):3):2,16:0):3):1,14:1):10):3);

tree PAUP_64 = [&R] (1:0,((2:2,3:2):0,(((((4:0,((5:0,9:2,10:0):1,(6:0,8:2):1):1,11:0):1,7:0,12:1):3,13:1):6,((15:2,(17:0,18:1):3):2,16:0):3):1,14:1):10):3);

tree PAUP_65 = [&R] (1:0,((2:2,3:2):0,(((((4:0,(((5:0,10:0):1,9:1):1,6:0,8:2):2,11:0):1,7:0,12:1):3,13:1):6,((15:2,(17:0,18:1):3):2,16:0):3):1,14:1):10):3);

End;

]

[Strict and Majority Rule consensus trees of the above 65 trees; remove brackets below to load trees]

[

Begin trees;

tree MajRule = [&U] (Protostomes,((Echinoderms,Enteropneusts),(((((Vetulicola,(Beidazoon,Nesonektris),Ooedigera),((Pomatrum_Xidazoon,Heteromorphus),((Didazoon,Yuyuanozoon),Banffia))),Tunicates),((Pikaia,(Vertebrates,Conodonts)),Yunnanozoans)),Cephalochordates)));

tree Strict = [&U] (Protostomes,((Echinoderms,Enteropneusts),((((Vetulicola,Pomatrum_Xidazoon,Didazoon,Beidazoon,Yuyuanozoon,Banffia,Heteromorphus,Ooedigera,Nesonektris),Tunicates),((Pikaia,(Vertebrates,Conodonts)),Yunnanozoans)),Cephalochordates)));

end;

]
