# Supplementary material for: A new vetulicolian from Australia and its bearing on the chordate affinities of an enigmatic Cambrian group
Source: BMC Evol Biol. 2014 Oct 21;14:214. doi: 10.1186/s12862-014-0214-z (PMC4203957; doi:10.1186/s12862-014-0214-z)
Supplement: Additional file 4: — Apomorphies in majority-rule consensus of the 65 most parsimonious trees. [file 12862_2014_214_MOESM4_ESM.doc]

**Additional file 4: Apomorphies in majority-rule consensus of the 65 most parsimonious trees.** This consensus is identical to one of the 65 primary trees.

Tree length = 58

Consistency index (CI) = 0.7586

Homoplasy index (HI) = 0.3793

CI excluding uninformative characters = 0.7455

HI excluding uninformative characters = 0.2545

Retention index (RI) = 0.7879

Rescaled consistency index (RC) = 0.5977

/------- Didazoon

/----23------- Yuyuanozoon

/-----24------------- Banffia

/-----25 /------- Pomatrum Xidazoon

| \-----------22------- Heteromorphus

/----26 /------- Beidazoon

| | /----20------- Nesonektris

/-----27 \------------21------------- Vetulicola

| | \------------- Ooedigera

| \--------------------------------- Tunicates

/-----31 /------- Vertebrates

| | /----28------- Conodonts

/----32 | /-----29------------- Pikaia

| | \------------------30-------------------- Yunnanozoans

/-----33 \----------------------------------------------- Cephalochordates

| | /------- Echinoderms

| \--------------------------------------------19------- Enteropneusts

\------------------------------------------------------------ Protostomes

Apomorphy lists:

Branch Character Steps CI Change

-------------------------------------------------------------------------------

Protostomes --> node_33 11 (11 Position o) 1 0.667 0 ==> 1

18 (18 Wide phari) 1 0.500 0 --> 1

19 (19 Pharingeal) 1 0.333 0 --> 1

node_19 --> Echinoderms 18 (18 Wide phari) 1 0.500 1 --> 0

19 (19 Pharingeal) 1 0.333 1 --> 0

node_19 --> Enteropneusts 24 (24 Suspension) 1 1.000 1 ==> 2

25 (25 Chordate p) 1 0.667 0 ==> 1

node_33 --> node_32 2 (2 Segmentation) 1 1.000 0 --> 1

4 (4 Myomeres) 1 1.000 0 ==> 1

7 (7 Body shape, ) 1 1.000 0 ==> 1

9 (9 Dorsal and o) 1 0.500 0 ==> 1

12 (12 Notochord) 1 1.000 0 ==> 1

14 (14 Dorsal ner) 1 1.000 0 ==> 1

15 (15 Median ven) 1 0.500 0 ==> 1

17 (17 Buccal cav) 1 0.500 0 --> 1

23 (23 Atrium) 1 0.333 0 --> 1

25 (25 Chordate p) 1 0.667 0 ==> 2

node_32 --> node_31 21 (21 Number or ) 1 0.500 2 --> 1

node_31 --> node_27 1 (1 Distinct ant) 1 1.000 0 ==> 1

6 (6 Thick cuticl) 1 1.000 0 ==> 1

8 (8 Whole body i) 1 1.000 1 ==> 0

16 (16 Position o) 1 1.000 1 ==> 0

19 (19 Pharingeal) 1 0.333 1 --> 0

27 (25 Notochord ) 1 1.000 0 ==> 1

node_27 --> node_26 11 (11 Position o) 1 0.667 1 ==> 0

17 (17 Buccal cav) 1 0.500 1 ==> 0

23 (23 Atrium) 1 0.333 1 --> 0

node_21 --> node_20 31 (31 Shape of a) 1 1.000 0 ==> 1

node_20 --> Nesonektris 32 (32 Shape of t) 1 0.500 0 ==> 1

node_26 --> node_25 29 (29 Orifice to) 1 1.000 1 --> 2

33 (33 Number of ) 1 0.500 0 --> 1

node_25 --> node_24 9 (9 Dorsal and o) 1 0.500 1 ==> 0

node_24 --> node_23 33 (33 Number of ) 1 0.500 1 --> 0

node_23 --> Yuyuanozoon 29 (29 Orifice to) 1 1.000 2 ==> 0

30 (30 Lateral gr) 1 1.000 1 ==> 0

node_24 --> Banffia 32 (32 Shape of t) 1 0.500 0 ==> 1

node_27 --> Tunicates 21 (21 Number or ) 1 0.500 1 --> 2

node_31 --> node_30 20 (20 Gills) 1 1.000 0 ==> 1

26 (26 Pre-oral s) 1 1.000 0 ==> 1

28 (28 Notochord ) 1 1.000 0 --> 1

node_30 --> node_29 23 (23 Atrium) 1 0.333 1 --> 0

24 (24 Suspension) 1 1.000 1 ==> 0

node_29 --> Pikaia 15 (15 Median ven) 1 0.500 1 ==> 0

20 (20 Gills) 1 1.000 1 ==> 2

node_29 --> node_28 5 (5 Myomere shap) 1 0.500 0 ==> 1

10 (10 Fin rays) 1 1.000 0 ==> 1

13 (13 Paired eye) 1 1.000 0 ==> 1

node_28 --> Conodonts 25 (25 Chordate p) 1 0.667 2 ==> 0

node_32 --> Cephalochordates 5 (5 Myomere shap) 1 0.500 0 --> 1
